# Serum Type XIX Collagen is Significantly Elevated in Non-Small Cell Lung Cancer: A Preliminary Study on Biomarker Potential

**DOI:** 10.3390/cancers12061510

**Published:** 2020-06-09

**Authors:** Jeppe Thorlacius-Ussing, Tina Manon-Jensen, Shu Sun, Diana J. Leeming, Jannie M. Sand, Morten Karsdal, Nicholas Willumsen

**Affiliations:** 1 Biomarkers and Research, Nordic Bioscience A/S, 2730 Herlev, Denmark; tmj@nordicbio.com (T.M.-J.); ssu@nordicbio.com (S.S.); djl@nordicbio.com (D.J.L.); jsa@nordicbio.com (J.M.S.); mk@nordicbio.com (M.K.); nwi@nordicbio.com (N.W.); 2 Department of Biomedical Sciences, University of Copenhagen, 2200 Copenhagen, Denmark

**Keywords:** Collagen XIX, ECM, biomarker, serum, cancer, NSCLC, AD, SCC

## Abstract

Type XIX collagen is a poorly characterized collagen associated with the basement membrane. It is abnormally regulated during breast cancer progression and the NC1 (XIX) domain has anti-tumorigenic signaling properties. However, little is known about the biomarker potential of collagen XIX in cancer. In this study, we describe a competitive ELISA, named PRO-C19, targeting the C-terminus of collagen XIX using a monoclonal antibody. PRO-C19 was measured in serum of patients with a range of cancer types and was elevated in non-small cell lung cancer (NSCLC) (*p* < 0.0001), small cell lung cancer (*p* = 0.0081), breast (*p* = 0.0005) and ovarian cancer (*p* < 0.0001) compared to healthy controls. In a separate NSCLC cohort, PRO-C19 was elevated compared to controls when evaluating adenocarcinoma (AD) (*p* = 0.0003) and squamous cell carcinoma (SCC) (*p* < 0.0001) patients but was not elevated in chronic obstructive pulmonary disease patients. SCC also had higher PRO-C19 levels than AD (*p* = 0.0457). PRO-C19 could discriminate between NSCLC and healthy controls (AUROC:0.749 and 0.826 for AD and SCC, respectively) and maintained discriminatory performance in patients of tumor stages I+II (AUROC:0.733 and 0.818 for AD and SCC, respectively). Lastly, we confirmed the elevated type XIX collagen levels using gene expression data from The Cancer Genome Atlas (TCGA) and Genotype-Tissue Expression (GTEx) initiatives. In conclusion, type XIX collagen is released into circulation and is significantly elevated in the serum of cancer patients and PRO-C19 shows promise as a cancer biomarker.

## 1. Introduction

Lung cancer is the most commonly diagnosed cancer and is the leading cause of cancer death [1]. Non-small cell lung cancer (NSCLC) represents approximately 85% of all lung cancer cases, wherein adenocarcinoma (AD) and squamous cell carcinoma (SCC) are the most common subtypes [2,3]. The majority of lung cancer cases are diagnosed in later stages, resulting in a grim overall five-year survival rate of 19% [4]. However, for patients diagnosed in the localized stages, where most of the patients can benefit from surgical resection, the five-year survival rate is 56% [2,4]. Early detection is therefore one of the primary ways to improve survival for lung cancer patients.

The tumor microenvironment is intricately involved in the traditional cancer hallmarks that dictate tumor progression [5]. One of the major components of the tumor microenvironment is the extracellular matrix (ECM), the non-cellular part of tissues, which influences virtually all of these cancer hallmarks [6]. The most dominant proteins in the ECM are the collagens, of which there are 28 different types [7]. Our research group has previously demonstrated that collagens and other ECM proteins can be valuable as non-invasive biomarker tools in different cancer forms [8,9,10,11,12,13,14,15,16,17].

Type XIX collagen is a minor collagen that comprises three α1 chains forming a 400 kDa homotrimer. Each chain comprises five collagenous triple helix domains interspersed with six non-collagenous domains. Based on its primary sequence, type XIX collagen belongs to the Fibril-Associated Collagen with Interrupted Triple helices family of collagens which mediate interactions between fibrillar collagens and other ECM components [18,19,20].

A description of expression and localization of type XIX collagen in the human lungs is lacking in the literature, but in other tissues localization is associated with the vascular-, neural-, muscular- and some epithelial basement membrane zones (BMZ) [21]. Interestingly, protein staining of type XIX collagen in the epithelial BMZ of breast carcinoma is partially lost in localized ductal carcinoma and is completely absent in invasive carcinomas. This loss occurs earlier than the loss of type IV collagen and laminin, suggesting that decreased type XIX collagen levels is a result of the early stages of BMZ remodeling [22].

Similar to the release of matrikines from the NC1 domains of type IV, XV and XVIII collagens, the C-terminal NC1 domain of type XIX collagen can be cleaved off and released. The resulting peptide can inhibit the growth and angiogenesis of melanoma in vivo and inhibit invasiveness in vitro [23]. The NC1 domain is cleaved off by the plasmin protease and interacts with αvβ3 integrin to inhibit the FAK/PI3K/Akt/mTOR signaling pathway as well as inhibit GSK3β phosphorylation [24,25].

In this study, we developed and validated an enzyme-linked immunosorbent assay (ELISA) to quantify the C-terminus of type XIX collagen in serum, named PRO-C19. We demonstrate the biological relevance and biomarker potential of the resulting assay in serum of cancer patients. We found that PRO-C19 was significantly elevated in the serum of several different cancer types. PRO-C19 could discriminate between NSCLC and healthy controls and showed promise as a biomarker for NSCLC.

## 2. Results

### 2.1. PRO-C19 ELISA Development

**Figure 1:**
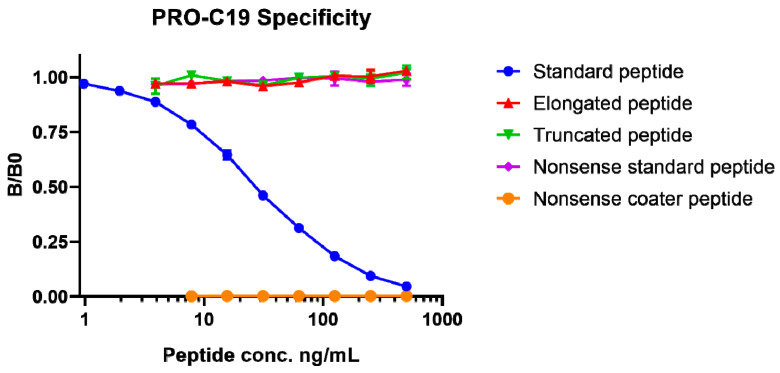
PRO-C19 assay specificity. Inhibition curve for the standard peptide (SHAHQRTGGN), elongated peptide (SHAHQRTGGNA), truncated peptide (SHAHQRTGG) as well as non-sense standard peptide (GVAPGIGPGG) and a non-sense coater peptide (Biotin-GVAPGIGPGG). Peptides were diluted twofold in series to evaluate their propensity to compete for antibody binding. Signal is shown as a fraction of the background absorbance (B0), corresponding to assay buffer, as a function of the peptide concentration on a logarithmic scale. Error bars indicate standard deviation from duplicate measurements.

Specificity of the competitive ELISA, named PRO-C19, was assessed by the proficiency of peptides to compete for binding to the monoclonal antibody. Peptides that were tested included the standard calibrator peptide (SHAHQRTGGN), an elongated calibrator peptide (SHAHQRTGGNA), a truncated calibrator peptide (SHAHQRTGG), a nonsense peptide (GVAPGIGPGG) and a nonsense coater peptide (Biotin-GVAPGIGPGG). Only the standard peptide dose-dependently inhibited the signal (Figure 1). The non-sense coater peptide resulted in no detectable signal. In all, this indicates that the assay is specific to the SHAHQRTGGN epitope of type XIX collagen.

**Figure 2:**
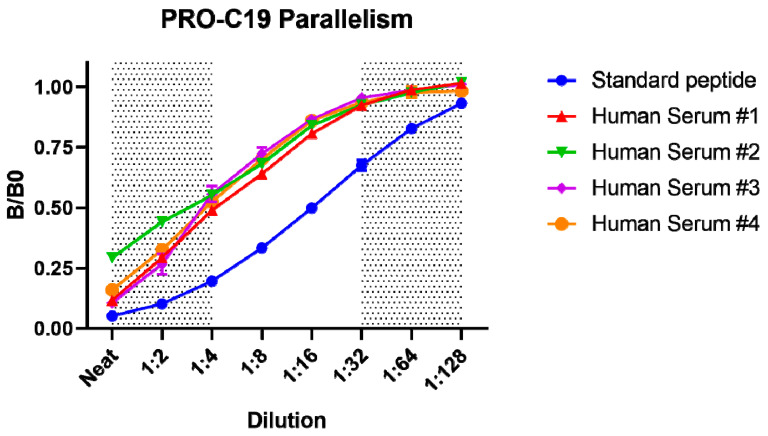
PRO-C19 parallelism. Inhibition curve for the standard peptide and human serum samples diluted twofold to assess linearity of dilution or parallelism. Signal is shown as a fraction of the background absorbance (B0), corresponding to assay buffer, as a function of the dilution steps. Error bars indicate standard deviation from duplicate measurements and greyed-out region indicates the limits of the linear range of the assay.

Technical validation of the PRO-C19 assay is summarized in Table 1. Linearity of dilution and parallelism was acceptable once serum samples were diluted 1:4, after which there was an average dilution recovery of 101.7% (Figure 2). Matrix accuracy in serum was acceptable with an average spiking recovery of 118.6% using the standard peptide spiked into human serum samples at a final dilution of 1:4. The influence of commonly interfering agents including hemoglobin, lipids and biotin was not observed. Inter-assay variation was 10.9% and intra-assay variation was 6.6%. The measurement range was determined as 3.31–214.3 ng/mL and the lower limit of detection was 1.23 ng/mL and upper limit 443.5 ng/mL. Analyte stability was acceptable for up to 24 h at 4 °C and up to 4 h at 20 °C. Freeze-thaw stability was acceptable for over 4 freeze-thaw cycles.

### 2.2. PRO-C19 in Serum of Cancer Patients (Cohort 1)

**Figure 3:**
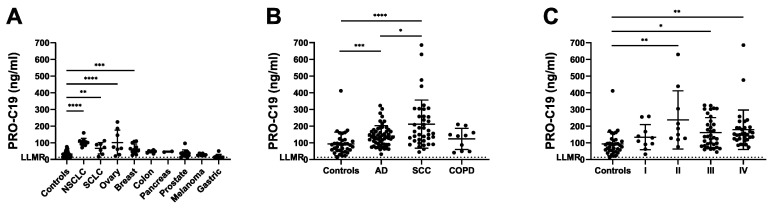
PRO-C19 in cohort 1 (A) and 2 (B and C). Quantification of PRO-C19 in the serum of: (**A**) healthy controls (*n* = 38) and a range of cancer types including NSCLC (*n* = 11), ovary (*n* = 8), SCLC (*n* = 7), breast (*n* = 12), colon (*n* = 7), pancreas (*n* = 2), prostate (*n* = 13), melanoma (*n* = 6), gastric (*n* = 9). (**B**) healthy controls (*n* = 35), AD (*n* = 55), SCC (*n* = 39) and COPD (*n* = 10). (**C**) healthy controls (*n* = 35), stage I (*n* = 10), stage II (*n* = 10), stage III (*n* = 35) and stage IV (*n* = 32). PRO-C19 levels are presented with mean and standard deviation. Samples measuring below the lower limit of measurement range (LLMR) were given the value of the LLMR, as determined in the validation of PRO-C19. Differences in PRO-C19 levels were evaluated by log-transforming the data and performing ordinary one-way ANOVA corrected for multiple comparisons with Tukey test. **** indicates a *p*-value below 0.0001. *** indicates a *p*-value below 0.001. ** indicates a *p*-value below 0.01. * indicates a *p*-value below 0.05.

As a first step to explore the usefulness of PRO-C19 in a cancer context, PRO-C19 was assessed in a cohort consisting of a range of cancer types including 12 breast cancer patients, 7 colon cancer patients, 9 gastric cancer patients, 6 melanoma patients, 11 NSCLC patients, 8 ovarian cancer patients, 2 pancreatic cancer patients, 13 prostate cancer patients, 7 small cell lung cancer (SCLC) patients as well as 38 healthy controls (Table 2). In the cancer group there was no significant association between PRO-C19 levels and age, BMI or smoking history. PRO-C19 levels were significantly elevated in NSCLC (*p* < 0.0001), SCLC (*p* = 0.0081), breast (*p* = 0.0005) and ovarian cancer (*p* < 0.0001) compared to healthy controls (Figure 3A). Although not significant, colon and pancreatic cancer groups also had higher mean PRO-C19 levels whereas gastric cancer had lower mean PRO-C19 levels as compared to healthy controls. With a cut-off of 63.3 ng/mL, PRO-C19 could discriminate between healthy and NSCLC with an area under receiver operating characteristic (AUROC) of 0.995 with a corresponding sensitivity of 100% and specificity of 94.74% (Table 3). PRO-C19 could also discriminate between healthy and SCLC with an AUROC of 0.808 at a cut-off of 54.3 ng/mL with a corresponding sensitivity of 71.4% and specificity of 84.2%. PRO-C19 could also discriminate between healthy and breast cancer with an AUROC of 0.814 at a cut-off of 41.85 ng/mL with a corresponding sensitivity of 75% and specificity of 78.9%. Lastly, PRO-C19 could also discriminate between healthy and ovarian cancer with an AUROC of 0.839 at a cut-off of 60.31 ng/mL with a corresponding sensitivity of 75% and specificity of 92.1%. Overall, although this cohort has its limitations, these results suggest that circulating levels of type XIX collagen are elevated in several different cancer types.

### 2.3. PRO-C19 in Serum of NSCLC Patients (Cohort 2)

Based on cohort 1 results, we decided to explore the role of PRO-C19 in NSCLC further. We therefore assessed PRO-C19 in an independent cohort of NSCLC patients including 55 AD patients and 39 SCC patients. The cohort also included 35 healthy controls and 10 chronic obstructive pulmonary disease (COPD) patients for comparison. For the AD group, 5 were stage I, 4 stage II, 22 stage III and 19 stage IV. For the SCC group, 5 were stage I, 6 stage II, 13 stage III and 13 stage IV (Table 2). Although there were slight variations in the age and gender proportions of the compared groups, we saw no association between PRO-C19 levels and age, gender, date of sample collection, BMI, smoking history, tumor grade, FEV1 or FEV1/FVC.

PRO-C19 levels were significantly elevated (*p* = 0.0003) with mean levels up to 1.5-fold higher for the AD group compared to controls (Figure 3B). A greater increase was seen in the SCC group, where mean PRO-C19 was significantly elevated (*p* < 0.0001) up to 2.25-fold higher compared to controls. Interestingly, the SCC group measured significantly higher than AD (*p* = 0.0457) with a 1.5-fold higher mean PRO-C19 level. Contrastingly, although the COPD group measured slightly above the controls, this group was not significantly different than controls, AD or SCC. To evaluate PRO-C19 in relation to severity of disease we divided cohort 2 into TNM stages. PRO-C19 levels trended upwards in later stages (not significant) and was significantly elevated in stages II, III and IV compared to controls (Figure 3C). Overall, these results confirm that PRO-C19 levels are elevated in the circulation of NSCLC patients.

In terms of diagnostic accuracy, PRO-C19 could discriminate between AD and controls with an AUROC of 0.749 with a corresponding sensitivity of 69% and specificity of 74% at a cut-off of 108.5 ng/mL (Table 3). Comparatively, discrimination between SCC and controls yielded an AUROC of 0.826 with a sensitivity of 72% and specificity of 83% at a cut-off of 124.1 ng/mL. Interestingly, PRO-C19 could also discriminate between the AD and SCC groups with an AUROC of 0.654 with a sensitivity of 41% and specificity of 87% at a cut-off of 194 ng/mL. Discrimination between COPD and the other groups was only significant for SCC with an AUROC of 0.690 with a sensitivity of 100% and specificity of 36%. PRO-C19 could also discriminate between stages I+II and controls for both NSCLC subtypes: for AD stages I+II yielded an AUROC of 0.733 and for SCC an AUROC of 0.818.

### 2.4. COL19A1 Gene Expression in Publicly Available Lung Cancer Databases

**Figure 4:**
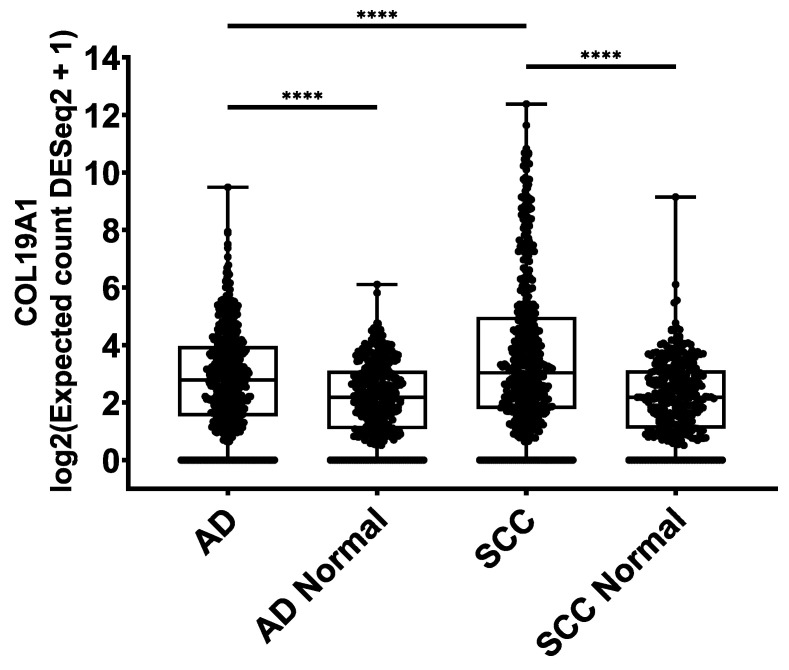
COL19A1 gene expression from the The Cancer Genome Atlas (TCGA) and Genotype-Tissue Expression (GTEx) databases in AD *(n* = 513), AD normal (*n* = 347), SCC (*n* = 498) and SCC normal (*n* = 338). The AD and SCC normal groups included normal samples from GTEx and tumor-adjacent normal samples from AD and SCC patients, respectively.

To support the relevance of type XIX collagen in lung cancer, we investigated the COL19A1 gene expression levels in normal lung and lung cancer using publicly available data from The Cancer Genome Atlas (TCGA) [26] and Genotype-Tissue Expression (GTEx) initiatives [27]. The normal lung dataset included 347 samples for AD and 338 for SCC, each comprising normal samples from the GTEx dataset and tumor-adjacent normal lung tissue from the TCGA AD and SCC datasets. The NSCLC dataset included 513 AD samples and 498 SCC samples. Median COL19A1 expression was significantly elevated in AD (*p* < 0.0001) and SCC (*p* < 0.0001) patients compared to their respective normal groups (Figure 4). SCC was also significantly elevated compared to AD (*p* < 0.0001). These data are in agreement with our assessment of PRO-C19 levels in circulation.

## 3. Discussion

The current study demonstrates the technical validation of an ELISA measuring the C-terminus of type XIX collagen named PRO-C19. PRO-C19 was specific towards the intended epitope and was technically robust. PRO-C19 was assessed in a panel of serum samples from healthy individuals, cancer patients and COPD patients to demonstrate biological relevance and biomarker potential. PRO-C19 levels were elevated in several types of cancer and could discriminate between NSCLC and healthy individuals with significant diagnostic accuracy in early stages of NSCLC.

To our knowledge, a description of type XIX collagen in the human lung is lacking and the presence of type XIX collagen in connection to lung cancer has not been demonstrated. Hence, this is the first study to demonstrate an association between lung cancer and type XIX collagen. Type XIX collagen has been observed in moderate amounts in the lungs of mice embryos, whereas only trace amounts were seen in adults, which could suggest a developmental role of type XIX collagen in the lungs [28]. Such an expression pattern is seen in several proteins and pathways important for cancer progression, and indeed several aspects of the developmental process are reactivated during tumorigenesis, including epithelial-mesenchymal transition [29,30,31]. Thus, a role in development could hint at a role in cancer as well.

PRO-C19 was excellent at discriminating between controls and some of the different cancer types, in particular NSCLC. As accuracy was also good in earlier stages of disease this could suggest that elevated levels of PRO-C19 may arise early in disease progression, potentially only in a subset of patients. Furthermore, future studies into early detection could also assess PRO-C19 in high-risk individuals before an eventual NSCLC diagnosis. Based on our limited data from COPD patients, we saw no elevated PRO-C19 levels, suggesting that PRO-C19 is not associated with lung disorders in general, but could instead be a more cancer-specific characteristic. A follow-up study further assessing PRO-C19 in COPD and other lung disorders such as idiopathic pulmonary fibrosis would be needed to confirm this. Our research group has developed several other ECM protein markers that have shown biological relevance in lung cancer [11,32,33,34,35,36,37,38]. Future studies should investigate combining PRO-C19 with these markers and other NSCLC biomarkers to improve overall accuracy.

In human adults, type XIX collagen expression can be very limited as exemplified by the 10^−6^% of the dry weight of umbilical cord tissue that type XIX collagen amounted to [39]. However, a separate study quantifying type XIX collagen in different tissue extracts and biological fluids found it detectable in the circulation [40]. Based on our data, type XIX collagen is released into circulation of healthy adults in modest amounts and circulating type XIX collagen levels are significantly increased in some cancer types. Type XIX collagen has previously been linked to breast cancer progression where, as the BMZ surrounding breast tumors was broken down during cancer progression, the staining of type XIX collagen protein was lost [22]. Based on our very limited data, an increase in the levels of circulating type XIX collagen is also linked to breast cancer. Type XIX collagen expression is strongly associated with the BMZ in general and the breakdown of the epithelial and vascular BMZ of the breast could lead to the release of type XIX collagen into circulation. It is important to note that there can be distinct differences in the organization of the BMZ of different tumor types e.g., the epithelial BMZ is broken down around invasive carcinomas of the breast, whereas it can remain intact around invasive glands in epithelial malignancies [22,41].

Anti-tumor properties have been assigned to type XIX collagen. The NC1 domain can, once cleaved off, inhibit invasion and angiogenesis in melanoma [25]. This was demonstrated in an in vivo mouse model where the NC1 (XIX) peptide inhibited tumor growth, and where the NC1 (XIX) peptide inhibited angiogenesis by matrix metalloproteinase-14 and vascular endothelial growth factor inhibition [23]. It was later discovered that NC1 (XIX) signaling is likely mediated by the αvβ3 integrin [25]. In a separate study, it was demonstrated that the NC1 (XIX) peptide could promote the formation of inhibitory nerve terminals through α5β1 integrin [42]. These integrin receptors are expressed by both epithelial and endothelial lung cells and can play a role in NSCLC, so it would be interesting to see the effects of NC1 (XIX) peptides in lung cancer [43,44,45]. Based on the peptides described in the literature, PRO-C19 is not specific towards the neo-epitope generated during plasmin cleavage of the NC1 domain. However, it can quantify any fragment containing the C-terminal epitope. Knowledge of how type XIX collagen is cleaved or otherwise processed is lacking, so PRO-C19 could hypothetically measure a large and diverse population of type XIX collagen fragments that all contain the C-terminal epitope. Further investigation into how type XIX collagen is processed and if any specific fragments can be quantified in circulation is warranted. Type XIX collagen has also been linked to neurodegenerative diseases including amyotrophic lateral sclerosis and Parkinson’s. Type XIX collagen expression is downregulated in the peripheral blood of Parkinson’s patients [46]. Contrastingly, in amyotrophic lateral sclerosis, type XIX collagen increased with progressing disease and increased mortality risk [47,48,49]. This is another avenue where the PRO-C19 assay could prove useful.

This study has several major limitations: Given the strictly exploratory nature of this study, the use of so-called “samples of convenience” and post-hoc analysis can introduce bias. In numbers, this bias is evidenced by the differences in sample sizes, age and gender of the compared groups. However, we did not observe any connection with PRO-C19 levels and these parameters in our limited dataset. Clinical data of the study participants was also limited, so additional hidden bias could also arise. Importantly, a study with substantially larger sample sizes is necessary to explore where PRO-C19 could prove useful in the clinical setting. The results of this study are therefore merely our first attempt at probing the biology of type XIX collagen in cancer. A limitation of our approach to type XIX collagen quantification is that the source tissue cannot be determined, although the tissue the tumor is found in is the likely contributor. Overall though, how the breakdown of the BMZ and circulating type XIX collagen are connected warrants further investigation. To support our findings using the PRO-C19 assay, we used COL19A1 gene expression data from the TCGA and GTEx initiatives to confirm the elevated levels of type XIX collagen in NSCLC. We saw elevated levels for AD and SCC compared to their respective control groups and COL19A1 levels in SCC were significantly higher than in AD. This is in accordance with the PRO-C19 data described above.

## 4. Materials and Methods

### 4.1. PRO-C19 ELISA Protocol

The ten amino acid peptide ^1133^SHAHQRTGGN^1142^ found in the very C-terminus of type XIX collagen (UniProtKB: Q14993) was purchased from Genscript (Piscataway, NJ, USA) and used for immunization. The production of monoclonal antibodies has been described elsewhere [32]. Several optimizations were made to the ELISA including the choice of assay buffer, incubation time and temperature as well as concentrations of antibody and peptides. The final PRO-C19 protocol was performed as follows: a 96-well streptavidin-coated ELISA plate was coated with 100 µl/well of 2.5 ng/mL biotinylated SHAHQRTGGN peptide dissolved in assay buffer (25 mM Tris-buffered saline, 1% bovine serum albumin (w/v), 0.1% Tween-20 (w/v), 2 g/l NaCl, pH 8.0) and incubated for 30 min at 20 °C with shaking at 300 revolutions-per-minute (RPM). After washing five times with washing buffer (25 mM Tris, 50 mM NaCl, pH 7.2), 20 µl/well of sample was added in duplicates followed by 100 µl/well of 60 ng/mL Horseradish peroxidase-labelled monoclonal antibody in assay buffer and incubated for 1 h at 20 °C with shaking at 300 RPM. After a second washing cycle, 100 µl/well of 3,3’,5,5’-tetramethylbenzidine was added and incubated 15 min in darkness at 20 °C with shaking at 300 RPM. The reaction was stopped by adding 100 µl/well of 1% H_2_SO_4_. Absorbance was measured at 450 nm with 650 nm as reference. To generate a standard curve, 20 µl/well of 500 ng/mL SHAHQRTGGN peptide, serially diluted twofold, was added to appropriate wells and a four-parametric mathematical fit was used to generate the curve. Each plate included 5 quality control samples comprising one human serum, one horse serum, one bovine cartilage explant and two peptide-in-assay-buffer samples to monitor intra- and inter-assay variation.

### 4.2. Technical Validation of the PRO-C19 ELISA

Antibody specificity was tested by the inhibition of signal by twofold dilutions of the standard peptide (SHAHQRTGGN), elongated peptide (SHAHQRTGGNA), truncated peptide (SHAHQRTGG) as well as non-sense standard peptide (GVAPGIGPGG) and a non-sense coater peptide (Biotin-GVAPGIGPGG). Linearity or parallelism was tested by serially diluting human serum samples twofold and calculating the percentage recovery relative to the dilution. Accuracy was tested by spiking the standard peptide into a human serum sample and calculating the percentage recovery of the peptide in the spiked sample. The influence of commonly interfering substances including hemoglobin, lipids and biotin were evaluated by spiking human serum samples up to either a high or low concentration of the interfering agents (hemoglobin low = 2.5 mg/mL, high = 5 mg/mL; lipid low = 1.5 mg/mL, high = 5 mg/mL; biotin low = 3 ng/mL, high = 9 ng/mL). Impact of spiking with the interference agents was calculated as the percentage recovery of the spiked sample relative to the non-spiked sample. Assay variation was tested by ten independent runs using ten quality control samples run in double-determinations. Five of the quality control samples were human serum, one was horse serum, one was bovine cartilage explant and three were standard peptide in assay buffer of varying concentrations. Intra-assay variation was calculated as the mean coefficient of variance for the double determinations of each of the ten runs. Inter-assay variation was calculated as the overall coefficient of variance across the ten runs. Lower- and upper-limits of measurement range (LLMR and ULMR, respectively) were determined across the ten independent runs and denote the boundaries of the linear range of the standard curve. Analyte stability was determined for three human serum samples incubated at 4 or 20 °C for 2, 4, 24 or 48 h. Stability was calculated as the percentage recovery of the incubated sample relative to the control sample kept at -20 °C. Freeze-thaw stability was evaluated by freezing and thawing human serum samples up to 4 cycles. Stability was calculated as the percentage recovery of the thawed sample relative to the sample that underwent a single freeze-thaw cycle. Lower limit of detection was calculated as the mean concentration of 21 blank samples containing assay buffer with 3 standard deviations added. Upper limit of detection was calculated as the mean concentration of standard peptide corresponding to the highest concentration of the standard curve across the ten independent runs with 3 standard deviations subtracted.

### 4.3. Patient Samples

The first cohort was in part obtained from the commercial vendor Asterand (Detroit, MI, USA). It included serum from 75 cancer patients including breast cancer (*n* = 12), colon cancer (*n* = 7), gastric cancer (*n* = 9), melanoma (*n* = 6), NSCLC (*n* = 11), ovarian cancer (*n* = 8), pancreatic cancer (*n* = 2), prostate cancer (*n* = 13), SCLC (*n* = 7) along with 38 healthy controls in part from Asterand and in part from another study population (reg. no. KA99070gm) [50]. The second cohort was obtained from the commercial vendor Proteogenex (Los Angeles, CA, USA). It included 55 AD patients of which 5 were stage I, 4 stage II, 22 stage III and 19 stage IV. It also included 39 SCC patients of which 5 were stage I, 6 stage II, 13 stage III and 13 stage IV. It also included 10 COPD patients and lastly 35 healthy controls obtained from Proteogenex and BioIVT (Westbury, NY, USA). According to the vendors, sample collection was approved by an Institutional Review Board or Independent Ethical Committee and patients gave their informed consent (Protocol numbers PG-ONC 2003/1 and WIRB® Protocol #20161665). All investigations were carried out according to the Helsinki Declaration.

### 4.4. Analysis of Publicly Available Genomics Databases

The UCSC Xena browser (http://xena.ucsc.edu/) was used to access the TCGA (https://www.cancer.gov/tcga) and GTEx (https://www.gtexportal.org/) datasets. In the Xena browser we used the TCGA TARGET GTEx combined cohort and filtered down to TCGA and GTEx lung samples. We used RSEM expected count (DESeq2 standardized) UCSC Toil RNA-seq Recomputed data for COL19A1 gene expression. How this data has been processed and standardized has been described elsewhere [51,52,53,54]. We pooled together the GTEx normal lung data with either TCGA normal AD or SCC data to make two groups for comparison with the TCGA AD or SCC primary tumor data.

### 4.5. Statistics:

PRO-C19 levels were log (10) transformed and tested for normality by D’Agostino-Pearson omnibus test. Comparison of PRO-C19 levels across groups was done using ordinary one-way ANOVA corrected for multiple comparisons using Tukey test. Differences in age between groups was evaluated using Mann-Whitney test if comparing two groups and Kruskal-Wallis test if comparing more. Differences in gender was evaluated using Fisher’s exact test if comparing two groups and Chi-square test if comparing more. The correlation between PRO-C19 levels and BMI, age, smoking etc. was evaluated using linear regression. Diagnostic accuracy was tested by the AUROC curve. Sensitivity and specificity were determined at the estimated optimal cut-off value according to the Youden Index. A *p* value below 0.05 was considered significant. Asterisks indicate the following significance levels: *p < 0.05; **p < 0.01; ***p < 0.001; ****p < 0.0001. When doing multiple comparisons tests, multiplicity adjusted *p*-values are reported. Statistical analysis and graphs were done in GraphPad Prism (version 8.2 for Windows, GraphPad Software, San Diego, CA, USA, www.graphpad.com) and MedCalc (MedCalc Statistical Software version 18.11.6 (MedCalc Software bvba, Ostend, Belgium; https://www.medcalc.org; 2019).

## 5. Conclusions

In conclusion, we developed and validated an ELISA targeting the C-terminus of type XIX collagen, named PRO-C19. PRO-C19 was used to quantify type XIX collagen in the serum of cancer patients, where it was significantly elevated in several cancer types as compared to healthy controls. PRO-C19 was subsequently assessed in a separate NSCLC cohort where it was also significantly elevated and exhibited diagnostic accuracy in early stage NSCLC. Elevated levels of type XIX collagen in NSCLC patients were confirmed using publicly available gene expression data. In all, type XIX collagen shows potential as a cancer biomarker and further studies into its use are warranted.

## 6. Patents

A patent for the PRO-C19 assay is on file and is owned by Nordic Bioscience.

## Figures and Tables

**Table 1:** Summary of the technical validation of PRO-C19.

Test	Result
IC50	29.5 ng/mL
Measurement range	3.31–214 ng/Ll
Detection range	1.23–443 ng/L
Minimum required dilution in human serum	1:4
Dilution recovery of human serum below 1:4	102%
Spiking recovery of peptide in serum	119%
Interference hemoglobin, low/high conc.	95.1%/93.4%
Interference lipids, low/high conc.	104%/104%
Interference biotin, low/high conc.	101%/101%
Inter-assay variation	10.9%
Intra-assay variation	6.63%
Analyte stability (24 hrs 4 °C/4 hrs 20 °C)	85.3%/82.3%
Freeze-thaw stability up to four cycles	97.7%

**Table 2:** Demographics of the cohorts.

**Cohort 1**	**Healthy Controls (*n* = 38**)		**Cancers (*n* = 75**)		***p*-Value**
**Age, median (min-max**)	**71.5 (60–82**)		60 (30–84)		<0.0001
Male, n (%)	0 (0.00%)		39 (52.0%)		<0.0001
Tumour type and stage, n	-		Breast cancer (*n* = 12), II:10, III:2		-
			Colon cancer (*n* = 7), II:3, III:4		
			Gastric cancer (*n* = 9), I:4, II:1, III:3, IV:1		
			Melanoma (*n* = 6), II:5, III:1		
			NSCLC (*n* = 11), I:5, II:3, III:3		
			Ovarian cancer (*n* = 8), I:1, II:2, III:5		
			Pancreatic cancer (*n* = 2), Not available		
			Prostate cancer (*n* = 13), I:1, II:12		
			SCLC (*n* = 7), I:2, II:1, III:3, IV:1		
**Cohort 2**	**Healthy controls (*n* = 35**)	**AD (*n* = 55**)	**SCC (*n* = 39**)	**COPD (*n* = 10**)	***p*-Value**
Age, median (min–max)	60 (51–72)	62 (41–78)	62 (50–71)	54 (50–60)	<0.0001
Male, n (%)	21 (60%)	21 (42%)	27 (73%)	5 (50%)	0.0339
Tumour stage, n	-	I:5, II:4, III:22, IV:19	I:5, II:6, III:13, IV:13	-	-

Abbreviations: NSCLC, Non-small cell lung cancer; SCLC, small cell lung cancer; AD, adenocarcinoma; SCC, squamous cell carcinoma; COPD, chronic obstructive pulmonary fibrosis

**Table 3:** Summary of AUROC tests.

Test	AUROC (95%CI)	*p*-Value	Sensitivity, % (95%CI)	Specificity, % (95%CI)	Cut-Off (ng/mL)
**Cohort 1**					
Controls v NSCLC	0.995 (0.918 – 1.00)	<0.0001	100 (71.5 – 100.0)	94.74 (82.3 – 99.4)	63.3
Controls v SCLC	0.808 (0.663 – 0.910)	0.0048	71.4 (29.0 – 96.3)	84.2 (68.7 – 94.0)	54.3
Controls v breast cancer	0.814 (0.678 – 0.910)	<0.0001	75.0 (42.8 – 94.5)	78.95 (62.7 – 90.4)	41.9
Controls v ovarian cancer	0.839 (0.701 – 0.931)	0.0003	75.0 (34.9 – 96.8)	92.1 (78.6 – 98.3)	60.3
**Cohort 2**					
Controls v AD+SCC	0.781 (0.700 – 0.849)	<0.0001	73.4 (63.3 – 82.0)	74.29 (56.7 – 87.5)	108.5
Controls v AD	0.749 (0.647 – 0.835)	<0.0001	69.09 (55.2 – 80.9)	74.29 (56.7 – 87.5)	108.5
Controls v SCC	0.826 (0.721 – 0.905)	<0.0001	71.79 (55.1 – 85.0)	82.86 (66.4 – 93.4)	124.1
AD vs SCC	0.654 (0.548 – 0.749)	0.0096	41.03 (25.6 – 57.9)	87.27 (75.5 – 94.7)	194.0
Controls v COPD	0.646 (0.489 – 0.782)	0.1926	60.00 (26.2 – 87.8)	82.86 (66.4 – 93.4)	124.1
AD vs COPD	0.556 (0.428 – 0.680)	0.6251	30.00 (6.7 – 65.2)	98.18 (90.3 – 100.0)	49.1
SCC vs COPD	0.690 (0.542 – 0.814)	0.0377	100.00 (69.2 – 100.0)	35.90 (21.2 – 52.8)	210.2
Controls v AD I+II	0.733 (0.579 – 0.855)	0.0197	88.89 (51.8 – 99.7)	57.14 (39.4 – 73.7)	89.1
Controls v AD III+IV	0.756 (0.644 – 0.847)	<0.0001	68.29 (51.9 – 81.9)	77.14 (59.9 – 89.6)	113.9
Controls v SCC I+II	0.818 (0.677 – 0.916)	<0.0001	81.82 (48.2 – 97.7)	80.00 (63.1 – 91.6)	118.9
Controls v SCC III+IV	0.833 (0.716 – 0.916)	<0.0001	73.08 (52.2 – 88.4)	82.86 (66.4 – 93.4)	124.1

Abbreviations: AUROC, area under receiver operating characteristics
